# Neuroprotective Effects of Oxymatrine on PI3K/Akt/mTOR Pathway After Hypoxic-Ischemic Brain Damage in Neonatal Rats

**DOI:** 10.3389/fphar.2021.642415

**Published:** 2021-04-13

**Authors:** Wei Wei, Min Lu, Xiao-bing Lan, Ning Liu, Wei-ke Su, Alexandr V. Dushkin, Jian-qiang Yu

**Affiliations:** ^1^ Collaborative Innovation Center of Yangtze River Delta Region Green Pharmaceuticals, Zhejiang University of Technology, Hangzhou, China; ^2^ Department of Pharmacology, Ningxia Medical University, Yinchuan, China; ^3^ Ningxia Hui Medicine Modern Engineering Research Center and Collaborative Innovation Center, Ningxia Medical University, Yinchuan, China; ^4^ Institute of Solid State Chemistry and Mechanochemistry, Novosibirsk, Russia

**Keywords:** hypoxic-ischemic brain damage, oxymatrine, neuroprotective, autophagy, PI3K/AKT/mTOR

## Abstract

Oxymatrine (OMT), a quinolizidine alkaloid extracted from traditional Chinese herb *Sophora flavescens* Ait, has drawn attention because of its beneficial bioactivities against hypoxic–ischemic brain damage (HIBD). However, the underlying molecular mechanism remains unclear. In this study, we determined the *in vivo* and *in vitro* effects of OMT on seven-day old Sprague–Dawley rats with HIBD and in a rat model of primary hippocampal neuron oxygen glucose deprivation reoxygenation (OGD/R). This study was aimed to evaluate whether OMT exerted neuroprotective effects mediated by the (phosphatidylinositol 3-kinase/protein kinase B/mammalian target of rapamycin) PI3K/Akt/mTOR pathway after HIBD. Experimental results showed that the alkaloid significantly improved the early neurofunctional development, brain water content, abnormal pathological changes, and necrosis of neurons after HIBD. Moreover, OMT enhanced the cell viability and stabilized the mitochondrial permeability transition pore in the primary hippocampal neurons after OGD/R. OMT significantly decreased the autophagosome generation, elevated the expression of PI3K, Akt, and mTOR, and simultaneously reversed the mRNA expression of microtubule-associated protein 1-light chain 3 (LC3), Beclin-1, and sequestosomel (P62) induced by hypoxia and ischemia. However, these protective effects against HIBD could be suppressed when rapamycin, a specific inhibitor of mTOR, was included. Hence, the OMT exerted neuroprotective effects against HIBD by attenuating excessive autophagy by mediating the PI3K/Akt/mTOR pathway.

## Introduction

Neonatal perinatal asphyxia usually results in hypoxic–ischemic brain damage (HIBD), which is characterized by cerebral hypoxia and decreased or suspended cerebral blood flows. This HIBD condition, is closely related to acute death and subsequent life-long neurological deficits in neonates and accounts for approximately 23% of the annual neonatal deaths (Lawn et al., 2005; Martin et al., 2019). Although antenatal and neonatal cares have been advanced in the past several decades, the incidence of HIBD is approximately 26 cases per 1,000 live births in developing countries (Yıldız et al., 2017; Li et al., 2019). Hypothermia is currently the only established treatment option that decreases the risk of death and neurodevelopmental impairment in infants with HIBD (Jacobs et al., 2013), such as cerebral palsy, hearing loss and other neuromotor disorders, from 60 to 45% (Martin et al., 2019). However, approximately 50% of infants are still at risk of neurological sequelae and even death with hypothermia treatment (Edwards et al., 2010; Wu and Gonzalez, 2015).

Potential injurious factors of neonatal HIBD are complex and involve mitochondrial damage, excitotoxicity, oxidative stress, inflammation, apoptosis and autophagy (Douglas-Escobar and Weiss, 2015). Autophagy is a programmed cell death pattern of self-degradation in aging, damaged organelles and long-lifespan proteins. Neuronal autophagy plays an important role in hypoxia–ischemia (HI)-induced neuronal loss. This process is also a double-edged sword, that is moderate autophagy can maintain the homeostasis and function of neurons. By contrast, excessive autophagy may destroy organelles and cytosol, leading to the destruction of cellular structures and functions. Reducing excessive autophagy and increasing neuronal survival can be effective therapeutic targets for neonatal HI brain injury. The kinase cascade including phosphoinositide 3 kinase (PI3K), protein kinase B (Akt), and the mammalian target of rapamycin (mTOR) (PI3K/Akt/mTOR) signaling pathway, is the central cascade involved in cell transcription, translation, migration, metabolism, proliferation, and survival (Koh and Lo, 2015). As the only protective pathway of autophagy, this cascade is closely related to the regulation of autophagy. Several studies have demonstrated that the PI3K/Akt/mTOR signaling pathway could attenuate autophagy when it is activated (Liu et al., 2019). The neuroprotective effect of the PI3K/Akt/mTOR pathway has been widely studied in cerebral ischemia. Previous studies have also shown that the inhibition of autophagy exert neuroprotective effects by regulating the PI3K/Akt/mTOR signaling pathway (Koh and Lo, 2015; Huang et al., 2018).

Chinese medicinal herbs have been widely used in clinical treatment because of their multiple targeting capacity and protective benefits in traditional Chinese medicine. Oxymatrine (OMT), a quinolizidine alkaloid extracted from traditional Chinese herb *Sophora flavescens* Ait (Funaya and Haginaka, 2012; Panthati et al., 2012) (Chinese name “Kushen”), has been proved to vast pharmacological actions, such as anti-inflammation, antioxidant, antiviral, immunomodulation, and neuroprotection activities (Cui et al., 2011; Dong et al., 2013; Wang and Jia, 2014; Zhao et al., 2015). We also showed in our previous study that this alkaloid could ameliorate brain injury in hypoxic-ischemic neonatal rats (Huang et al., 2018). However, the molecular mechanism of neuroprotection by OMT remains unclear. Based on a study that reported the involvement of PI3K/Akt/mTOR in the autophagy processes in HIBD, we hypothesized that PI3K/Akt/mTOR pathways could be a part of the therapeutic effects of OMT. Therefore, we performed *in vivo* and *in vitro* experiments to explore deeply the protective effect of OMT after HIBD and determine its relationship with the PI3K/Akt/mTOR signaling pathway in regulating autophagy.

## Materials and Methods

### Animals and Establishment of the HIBD Model

Seven-day old Sprague–Dawley rats, 12–17 g weight (The ratio of male to female is 1:1) were provided by the Experimental Animal Center of Ningxia Medical University (animal license number: SCXK Ningxia 2015-0001). All surgeries and sample collection were carried out under diethyl ether anesthesia to minimize suffering and the number of animals used. The protocols were performed in accordance with the current guidance for the care of laboratory animals in Ningxia Medical University. All the researchers who performed testing were blinded.

A modified Rice-Vannucci model was established as previously described (Vannucci and Vannucci, 2005). Seven-day-old Postnatal 7 days (P7) neonatal Sprague–Dawley rats were narcotized by ether inhalation, the left common carotid artery was ligated with 6-0 silk and cut off within 5 min. After the surgery, the rats were allowed to return to their dam and recover for 1.5 h. Then, the pups were placed in a low-oxygen container (8% oxygen in nitrogen) maintained at 37°C and hypoxia for 2.5 h. All surviving pups were returned to their dam after 2.5 h of hypoxia. In the sham group, the left common carotid artery was only exposed. Our previous experiments showed that brain tissue suffers moderate damage at 48 h time point (Huang et al., 2018), we selected this time point for the following experiments.

### Drug Administration

OMT (Beijing Zhongke quality inspection Biotechnology Co., Ltd., China, purity = 98.26%, Batch NO: Y-013-161216) was dissolved in normal saline (NS; 0.9% NaCl) before the experiment. Drugs were administered intraperitoneally at 0.1 ml/10 g body weight. To determine the neuroprotective effects on HIBD, unsexed pups were assigned randomly into 3 groups (*n* = 6 for each group) as follows: Group 1, control (sham surgery) with an equal volume by body weight of NS; Group 2, subjected to cerebral hypoxia-ischemia (HI) with an equal volume by body weight of NS; Group 3, HI with OMT (120 mg/kg). OMT was intraperitoneally (ip) injected at 12 h intervals for 2 days. To further assess between the neuroprotective of OMT and the PI3K/Akt/mTOR pathway, the mTOR inhibitor rapamycin (MCE, America) were dissolved in 2% carboxymethylcellulose sodium (CMC-Na) and administered via intraperitoneal (ip) injection 1 h before HI (Bodine et al., 2001).

### Measurement of Neurobehavioral Development

According to previous published literature (Wu et al., 2017), the neurological reflexes including righting reflex, negative geotaxis reflex and cliff avoidance reflex was elicited at 48 h after HI. An outside environment was maintained at 37°C, each reflex assessed subsequently, and the pups were given 5 min of rest.

### Determination of Infarct Volume

After 48 hours of HI, the brain infarct volume was measured according to the previously described (Wei et al., 2019). The infarct volumes were measured through image analysis software (Image-Pro Plus, United States). The brain infarction volume was calculated accurately as follows to exclude the influence of cerebral edema (Lin et al., 1993): The percentage of brain infarction (%) = (normal hemisphere volume − non-infarct volume of the infarct side)/normal hemisphere volume × 100.

### Measurement of the Brain Water Content

The cerebral water content according to the wet-dry method as previously described (Wang et al., 2018). Briefly, brains were removed quickly and acquired the wet weight using an electronic analytic balance. Then, the samples were dried in a 100°C oven for 24 h and weighed again to obtain the dry weight. The following formula was used to determine the degree of brain water content: water content (%) = (wet weight − dry weight)/wet weight × 100% (Zhu et al., 2018).

### Hematoxylin and Eosin (HE) Staining

After 48 h of HI, rats were anesthetized and perfused with ice NS followed by 4% cold paraformaldehyde. Then, the brains removed immediately and submerged in 4% paraformaldehyde for overnight. Every tissue specimen was dehydrated and embedded in paraffin, and then successive brain coronal sections (5 μm) made with a microtome (Leica, Germany). The sections were dried at 80°C for 30 min, deparaffinized, rehydrated and stained with hematoxylin and eosin. The histopathological changes in cerebral hippocampus CA1, hippocampus CA3 and cortex were observed with light microscopy (Olympus BX-51, Japan) at a magnification of 200× and 400×.

### Terminal Deoxynucleotidyl Transferase-Mediated dUTP Nick-End Labeling (TUNEL) Staining

The slices were deparaffinized, rehydrated and then permeated with proteinase K to increase cell membrane permeability. The DNA fragmentation of apoptotic or necrotic cells bound to the terminal deoxynucleotidyl transferase (TdT) enzyme in a reaction buffer. After rinsing with PBS thoroughly, slides were mounted with 4′, 6-diamidino-2-phenylindole (DAPI) for at room temperature for nuclear staining. Ultimately, the TUNEL-positive neurons in 6 cortical fields of each group were photographed randomly with an Olympus fluorescence microscope (Olympus FV1000, Japan) at a magnification of 400×. The following formula was used to calculate the rate of apoptosis: apoptotic neurons amount/total neurons amount × 100%.

### Measurement of Transmission Electron Microscopy (TEM)

The cerebral tissues were prefixed with ice fixative solution for 2 h, and cut into 1 mm × 1 mm × 1 mm blocks. They were collected and fixed for 2 h with 2% cold glutaraldehyde. Then, the blocks were post-fixed with 2% ice osmium tetroxide, dehydrated, and embedded in epon. Ultrathin sections (75 nm-thick) were cut, placed onto colloid-coated copper grids and stained with 0.4% uranyl acetate and 2% lead acetate. Finally, Samples were observed by TEM (Hitachi, Tokyo, Japan).

### Primary Hippocampal Neurons Culture and Oxygen Glucose Deprivation/Reoxygenation (OGD/R) Model

The primary hippocampal neurons were prepared from brains (within 24 hours of born) of SD rats. Briefly, the hippocampus from newborn rats were separated carefully and placed in D-Hanks' balanced salt solution. After trituration and digestion with trypsase (containing EDTA) for 15 min at 37°C, the cell suspensions were seeded at a density of 1 × 10^6^/L in 96-well plates or culture dishes in DMEM supplemented with 2% HEPES-buffered salt solution and 10% FBS. Afterward, DMEM culture medium was discarded and changed to neurobasal medium containing 2% B27. After that, change the culture medium every 2°days.

The primary cultured hippocampal neurons were subjected to OGD insult for 2 h and reperfusion for 24 h on day 7. Specifically, the culture medium was changed to glucose-free EBSS, a mixed gas (95% N_2_ and 5% CO_2_) was continuously pumped into the incubator for 2 h at 37°C. After the OGD, the culture medium was replaced with regular medium and put back into a normoxic incubator at normal conditions and OMT (5 μg/ml) with or without Rapamycin was added for 24 h. Rapamycin was dissolved in DMSO and the final concentration was 0.2 μg/ml. In the control group, the neurons were cultured under normal conditions without oxygen or glucose deprivation.

### MTT Assay Cell Viability

To determine whether OMT could ameliorate OGD/R-induced neuronal death, Methyl-thiazolyl-diphenyl-tetrazolium bromide (C0009, Beyotime, China) assays were used to evaluate the neuronal viability according to the manufacturer's instructions. Briefly, primary hippocampal neurons were plated in 96-well plates. After reoxygenation for 24 h, 20 μL of MTT solution was added and incubated at 37°C for 4 h. After incubation, the medium was replaced, and the neurons were suspended in dimethyl sulfoxide (DMSO). The optical density (OD) was detected at 490 nm by a microplate reader and the results are expressed as the percentages of cell viability compared to the control group.

### Determination of Mitochondrial Permeablity Transition Pore (mPTP)

Primary hippocampal neurons were underwent OGD/R 24 h later, the determination of mPTP according to the operation of mPTP detection kit. Diluting the dye solution to the desired concentration, and then added 1 ml of staining working fluid to each dish. The neurons were incubated with staining working fluid at 37°C for 15 min. After washing three times with PBS and then imaged with an Olympus fluorescence microscope (Olympus FV1000, Japan) at excitation wavelengths of 488 nm and emission wavelengths of 520 ± 10 nm, respectively.

### Monodansylcadaverine (MDC) Staining

After 24 h of OGD/R, neurons were centrifuged at 800 g for 5 minutes and collected after cleaning with 1 × Wash buffer (300 μL). Afterward, the supernatant was sucked out and suspended with equal volume 1 × Wash buffer, the concentration of neuron was adjusted to 10^6^/ml. The 90 μL cell suspension and 10 μL MDC dye solution was mixed evenly and stained at room temperature for 35 minutes. After centrifugation, the neurons were washed again with 300 μL 1×Wash buffer. Subsequently, Collection buffer was added to collect suspended neurons, which added to the slides and observed under an Olympus fluorescence microscope (Olympus FV1000, Japan) at a magnification of 600×.

### Immunofluorescence Analysis

For the immunofluorescence analyses, hippocampal neurons were washed with PBS three times and incubated with 4% paraformaldehyde for 15 min. Afterward, the cells were incubated in goat serum for 60 min at room temperature to block unrelated antigens. The primary anti-*p*-mTOR antibody (1:100, Cell signaling technology, 5536S) was applied to the cells at 4°C overnight and followed by incubation with Rhodamine (TRITC)–conjugated secondary antibody at 37°C for 1 h in the dark. Ultimately, the nuclei were stained with DAPI for 5 min and images were acquired with a fluorescence microscope (Olympus BX-51, Japan) and the fluorescence intensity was analyzed with the ImageJ software.

### Western Blot Analysis

The rats were euthanized by anesthesia with inhaling ether 48 h after HIBD, and the ischemic hemisphere were removed rapidly in ice and stored at −80°C. Primary hippocampal neurons were collected from the culture dishes 24 h after OGD/R. The ischemic hemisphere and primary hippocampal neurons were homogenized in ice-cold lysis buffer in glass homogenizers (Nanjing Jiancheng Bioengineering Institute, Nanjing, China) or epoxide tubes. The homogenate was centrifugated at 12,000 g for 10 min at 4°C to obtain the total protein. The protein concentration of the samples was analyzed by BCA method according to manufacturer’s instruction (Nanjing Jiancheng Bioengineering Institute, Nanjing, China). Equal amount of protein lysate (50 μg) in each simple were separated by 10% or 6% sodium dodecyl sulfate polyacrylamide gel electrophoresis (SDS-PAGE) and then transferred onto polyvinylidene fluoride (PVDF) membranes (200 mA, 2 h). Membranes was subsequently blocking with 5% skim milk powder for 2 h at room temperature and incubated with the primary antibodies overnight at 4°C (Table 1). After washing with PBST three times (containing 20% Tween-20), the PVDF membranes were incubated with the secondary antibodies (1:2000, SA00001-2; Proteintech) for 2 h at room temperature. The protein bands were observed by a Western blot detection system (Bio-Rad Laboratories, United States). Finally, the gray values of the bands were analyzed by Quantity One software.

**TABLE1:** The primary antibodies in the experiment.

Antibody	Company	Catalog number	Dilution multiple (*in vivo*/*in vitro*)
PI3K	Cell signaling technology	20584-1A	1:1,000/1:500
p-PI3K	Cell signaling technology	4228S	/1:500
Akt	Abcam	GR242901-30	1:2000/1:500
p-Akt	Cell signaling technology	9271S	/1:500
mTOR	Abcam	GR245538-8	1:1,000/
LC3	Proteintech	14600-1-AP	1:500/
Beclin-1	Proteintech	11306-1-AP	1:800/
P62	Proteintech	18420-1-AP	1:1,000/
β-actin	Proteintech	20536-1-AP	1:1,000/1:1,000

### Quantitative Real-Time Polymerase Chain Reaction (Q-PCR)

At 24 h after OGD/R, total RNA of primary hippocampal neurons was extracted in accordance with the previous description (Zhao et al., 2018). Total RNA was then reverse transcribed, amplified and real-time Q-PCR analysis. Ultimately, the PCR products were analyzed quantitatively by using the melting curve. The fold induction (2^-△△Ct^) was represented as the calculated results. Nucleotide sequences of primers utilized are as follows: Beclin1 (forward 5′-AGGAGTTGCCGTTGTACTGTTCTG-3′/reverse 5′-TGC​CTC​CAG​TGT​CTT​CAA​TCT​TGC-3′; 183 bp, 61.2°C), LC3 (forward 5′-AGC​TCT​GAA​GGC​AAC​AGC​AAC-3′/reverse 5′-GCT​CCA​TGC​AGG​TAG​CAG​GAA-3′; 101 bp, 59.8°C), P62 (forward 5′-GGT​GTC​TGT​GAG​AGG​ACG​AGG​AG-3′/reverse 5′-TCT​GGT​GAT​GGA​GCC​TCT​TAC​TGG-3′; 101 bp, 60.7°C) and β-actin (forward 5′-CCC​ATC​TAT​GAG​GGT​TAC​GC-3′/reverse5′-TCTGGTGATGGAGCCTCTTACTGG-3′; 174 bp, 60.0°C).

### Data Analysis

The apoptosis ratio was analyzed by nonparametric test. The other values were expressed as mean ± SEM, and the statistical analysis of the results was evaluated by one-way ANOVA followed by Dunett’s test. *p* < 0.05 was considered statistically significant.

## Results

### OMT Improved HIBD-Induced Neurological Dysfunction

**FIGURE 1:**
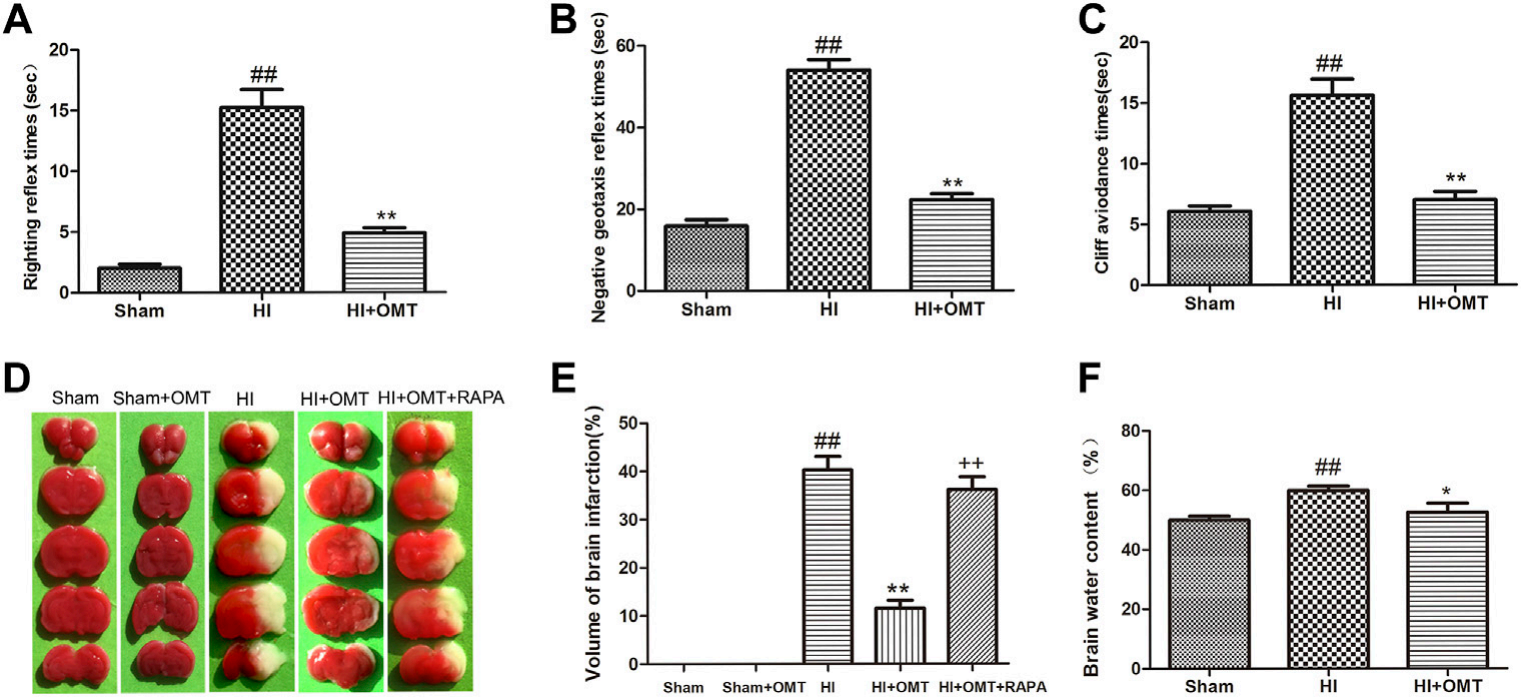
Changes in the early neurological reflex performances, infarct volume and water volumes in neonatal rats at 48 h after hypoxia-ischemia brain damage (HIBD). **(A)** The latency of righting reflex. **(B)** The latency of negative geotaxis reflex and **(C)** The latency of n Cliff avoidance reflex. D Representative photographs of the infarct volume identified with 2,3,5-triphenyltetrazoliuM chloride (TTC) staining. **(E)** Measured infarct volumes. **(F)** Assessments of brain water content. The results are expressed as means ± SEM (*n* = 6 per group). ^##^ *p* < 0.01 vs. the sham group; *p < 0.05, **p < 0.01 vs. the HI group; and ^++^ *p* < 0.01 vs. the oxymatrine (OMT) group.

Compared with the Sham group, the rats that were subjected to HI exhibited a prolonged latent period in righting reflex, negative geotaxis and cliff avoidance reflex (*p* < 0.01, Figures 1A–C). However, the increased time for above reflexes was reduced significantly (*p* < 0.01, Figures 1A–C) in the HI + OMT group than that in HI group.

### OMT Ameliorated HIBD-Induced Cerebral Infarction

2,3,5-Triphenyltetrazolium chloride (TTC) staining is a recognized indicator for evaluating the volume of cerebral infarction. As shown in the images of the TTC staining in Figure 1D, the infarct volume was not observed and stained uniformly red in the sham group. The total infarct volume was significantly reduced from 40.32 ± 6.15%–11.60 ± 3.54% (*p* < 0.01) in the HI + OMT group compared with that in the HI group (Figure 1E, *p* < 0.01). Rapamycin (RAPA) treatment markedly increased the cerebral infarct volume (*p* < 0.01) compared with the HI + OMT group.

### OMT Decreased HIBD-Induced Cerebral Edema

Brain water content was assessed to evaluate the brain edema (Figure 1F). Compared with the sham group, the brain water content increased significantly after cerebral ischemia and hypoxia (*p* < 0.01). However, post-administration with OMT attenuated the brain water content from 59.90 ± 3.20%–52.60 ± 6.61% compared with the HI group (*p* < 0.05).

### OMT Attenuated HIBD-Induced Neuron Pathological Changes and Apoptosis

**FIGURE 2:**
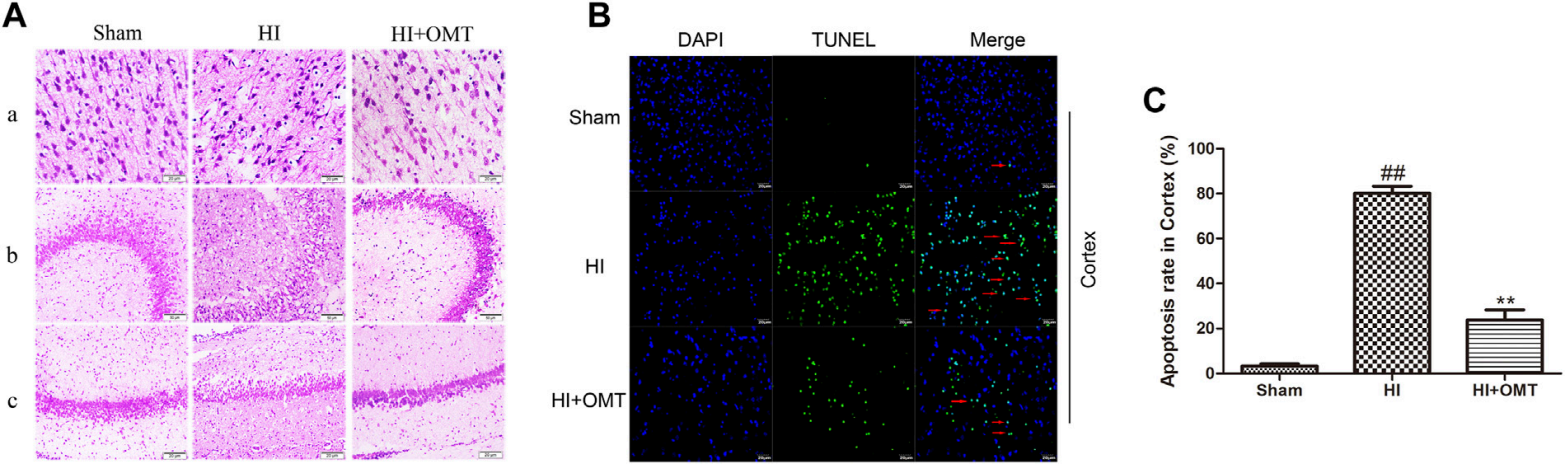
Representative photographs of the hematoxylin and eosin (HE) and terminal deoxynucleotidyl transferase-mediated dUTP Nick End Labeling (TUNEL) staining in the hippocampus and cortex for the damaged neurons in the neonatal rats at 48 h after hypoxia-ischemia brain damage (HIBD). **(A)** Representative photographs of the morphological changes identified by HE staining: **(A)** cortex, **(B)** hippocampus CA3, and **(C)** hippocampus CA1. **(B)** Representative photographs of the neuronal apoptosis identified by TUNEL staining. **(C)** Quantitative representation of the expression of apoptotic neurons in the cortex. The results are expressed as mean ± SEM (*n* = 6 per group). ^##^ *p* < 0.01 vs. the sham group; and **p < 0.01 vs. the hypoxia-ischemia (HI) group.

As shown in Figure 2A, neurons were abundant, arranged neatly and stained evenly in the cerebral hippocampus CA1, hippocampus CA3, and cortex in the sham group. Compared with the sham group, the number of cells decreased, the cells became arranged disorderedly, vacuolization, and karyopyknosis were observed in the HI group. After posttreatment with OMT, the expansion of neuronal injury was reversed to a certain extent in the cerebral hippocampus CA1, hippocampus CA3, and cortex (Figure 2A). The protective effect of OMT was further determined by TUNEL staining from the ischemic cortex. Virtually rare TUNEL-positive neurons were found in the sham group. By contrast, numerous TUNEL-positive neurons were detected in the HI group. However, compared with the HI group, the neuronal apoptosis was reduced substantially in the posttreatment with OMT (Figures 2B,C; *p* < 0.01).

### OMT Inhibited OGD/R-Induced Cell Death, the Openess of mPTP in Cultured Hippocampal Neurons

**FIGURE 3:**
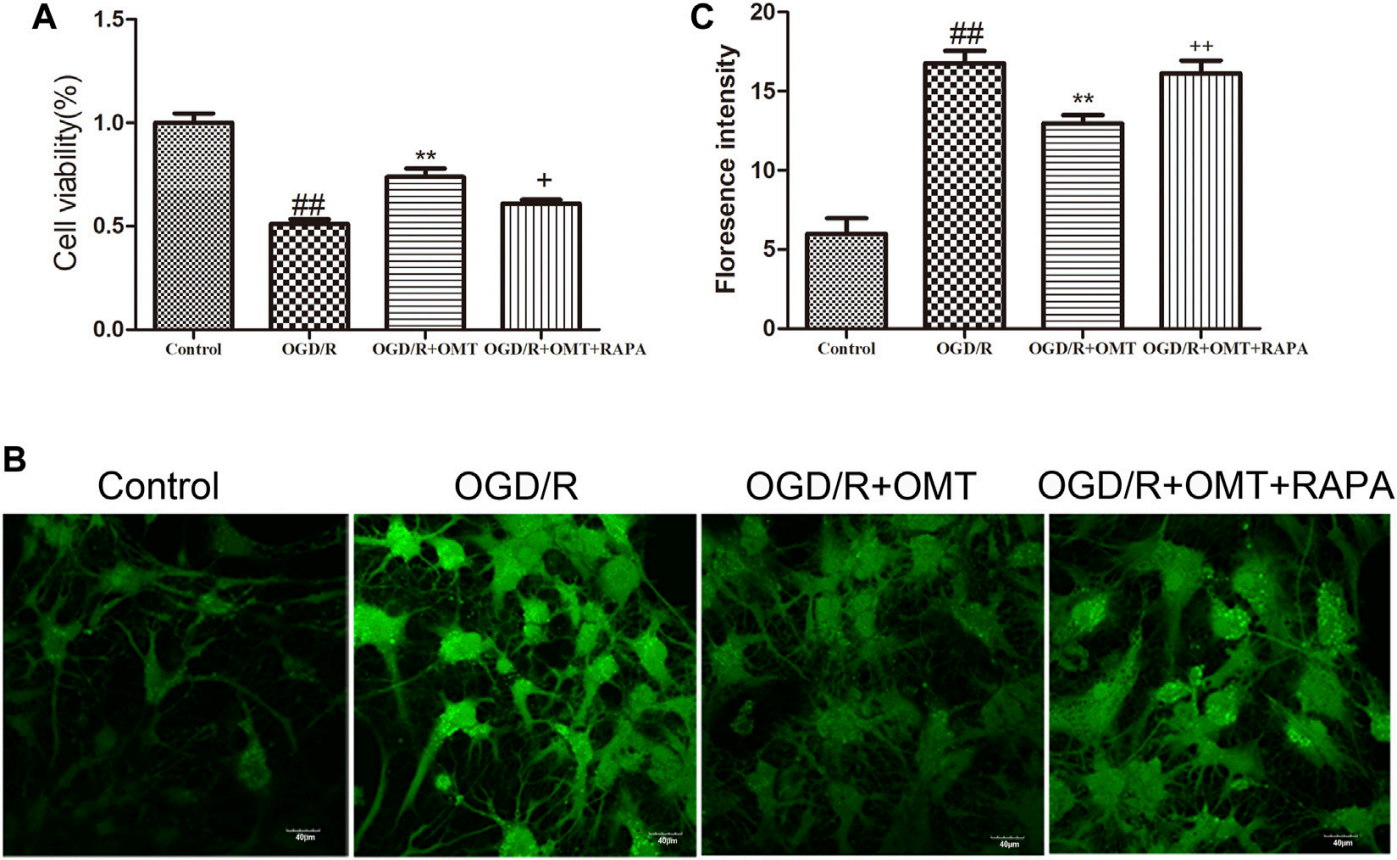
Assessments of neuronal death and level of intracellular mitochondrial permeability transition pore (mPTP) in the primary hippocampal neurons at 24 h after oxygen-glucose deprivation reperfusion (OGD/R). **(A)** Cell viability assay. **(B)** Fluorescence micrographs of mPTP. **(C)** mPTP assay. The results are expressed as mean ± SEM (*n* = 6 per group). ^##^ *p* < 0.01 vs. the sham group; **p < 0.01 vs. the HI group; and ^+^ *p* < 0.05, ^++^ *p* < 0.01 vs. the oxymatrine (OMT) group.

The results of the MTT assay for cell viability observed for 24 h after oxygen glucose deprivation reoxygenation (OGD/R) are shown in Figure 3A. Cell viability was significantly reduced in the OGD/R group compared with the control group (*p* < 0.01). Posttreatment with OMT (5 μg/ml) significantly enhanced the cell viability (*p* < 0.01) compared with that in the OGD/R group. The fluorescence intensity of the mPTP was consistent with that of cell viability. Interestingly, RAPA treatment reversed these effects of OMT (Figures 3A,C, *p* < 0.05, *p* < 0.01).

### OMT Reduced the Level of Autophagosome After HIBD and OGD/R

**FIGURE 4:**
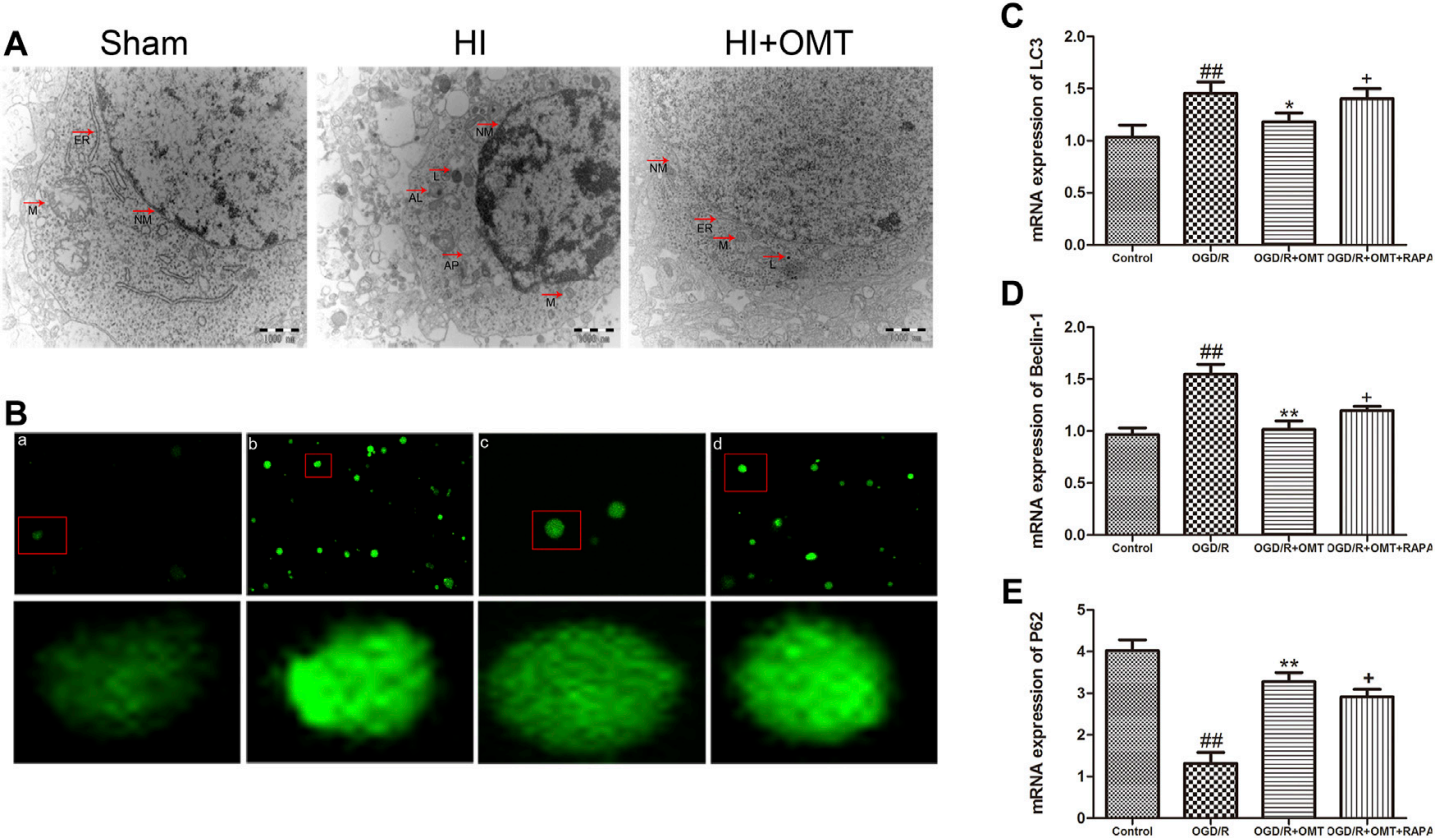
Assessments of the level of autophagy *in vivo* and *in vitro*. **(A)** Transmission electron micrographs of the autophagosomes in the neonatal rats at 48 h after hypoxia-ischemia brain damage (HIBD). The arrows represent organelles and autophagosome: N, nucleus; NM, nucleus membrane; M, mitochondrion; ER, endoplasmic reticulum; L, lysosomes; and AP, autophagosome. **(B)** Autophagic vacuoles from each group measured by monodansylcadaverine (MDC) staining. **(C**–**E)** Levels of microtubule-associated protein 1-light chain 3 (LC3), Beclin-1 and sequestosomel (P62) mRNA in the primary hippocampal neurons measured at 24 h after oxygen-glucose deprivation reperfusion (OGD/R). The results are expressed as means ± SEM (*n* = 6 per group). ^##^ *p* < 0.01 vs. the sham group; *p < 0.05, **p < 0.01 vs. the HI group; and ^+^ *p* < 0.05 vs. the oxymatrine (OMT) group.

We evaluated the level of autophagy formation in the brain tissue *in vivo* and *in vitro* by tissue ultrastructure and MDC staining, respectively (Figures 4A,B). As demonstrated in Figure 4, autophagosomes were identified in the HI group but not in the OMT treatment group and sham group. MDC positive staining increased in the primary hippocampal neurons after OGD/R compared with that in the control group. MDC positive staining decreased with post-administration with OMT. By contrast, posttreatment with RAPA increased the MDC positive staining.

### OMT Regulated the Expression of Autophagy-Related Protein and MRNA Induced HIBD and OGD/R

**FIGURE 5:**
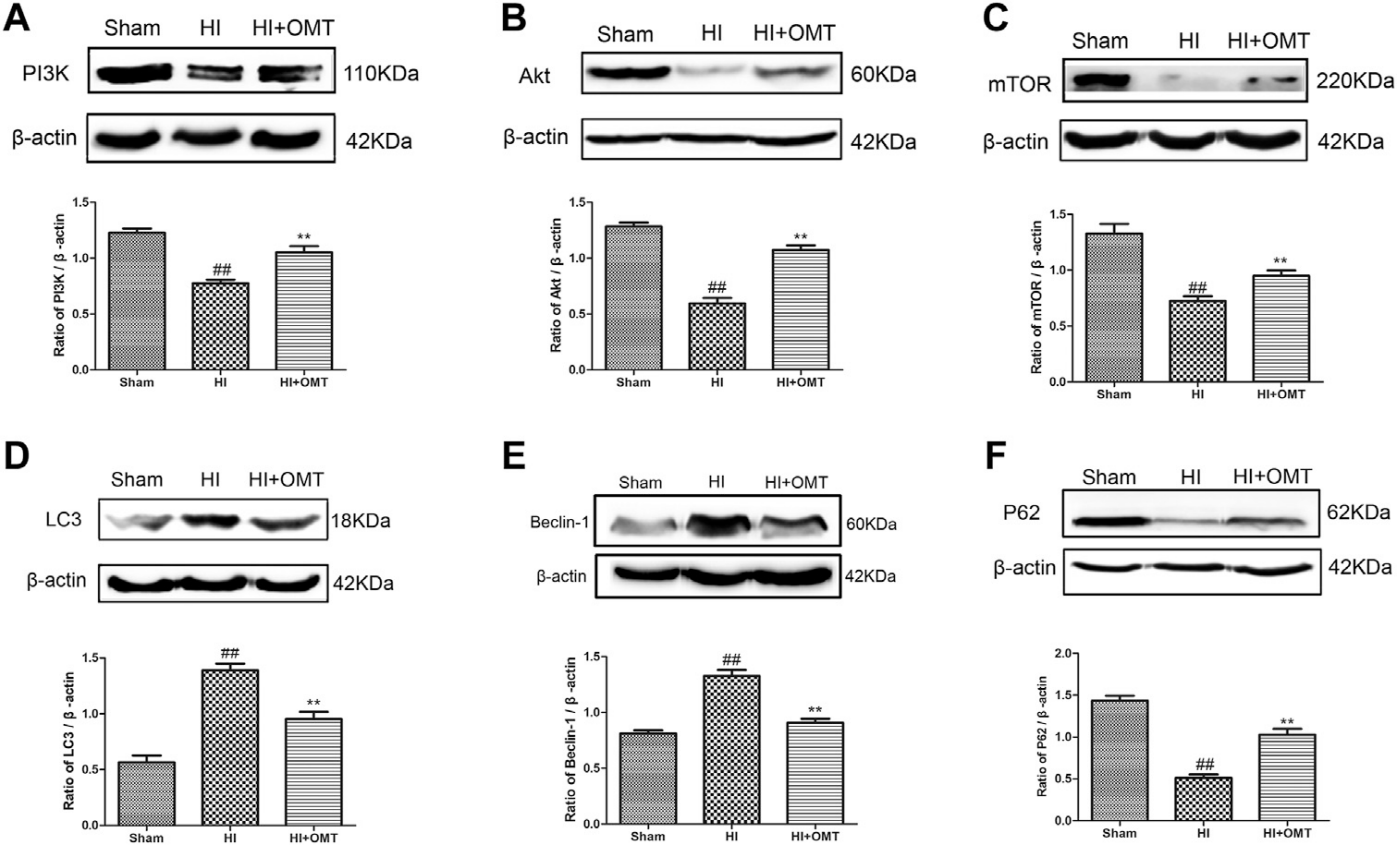
Assessment of the protein expression levels of phosphatidylinositol 3-kinase (PI3K), protein kinase B (Akt), mammalian target of rapamycin (mTOR), LC3, Beclin-1 and P62 in the ischemic hemisphere in the neonatal rats at 48 h after hypoxia-ischemia brain damage (HIBD). Representative Western blots and quantitative analyses of the protein expression of **(A)** PI3K, **(B)** Akt, **(C)** mTOR, **(D)** LC3, **(E)** Beclin-1 **(F)** and P62. The results are expressed as means ± SEM (*n* = 6 per group). ^##^ *p* < 0.01 vs. the sham group; and **p < 0.01 vs. the HI group.

We further tested the effects of OMT on the mRNA expression of autophagy-related factors, such as Beclin-1, LC3, and P62 protein. As shown in Figures 4C–E, the mRNA expression of Beclin-1 and LC3 was significantly increased in the OGD/R group than that in the control group (*p* < 0.01). Post-administration of OMT induced a substantial decrease in the mRNA expression of Beclin-1 and LC3 (*p* < 0.01, *p* < 0.05). The expression of P62 mRNA significantly decreased compared with that in the control group (*p* < 0.01), but, OMT treatment could upregulate markedly its expression (*p* < 0.01). In addition, the expression of the above autophagy-related factors in the OMT could be partially eliminated by RAPA (*p* < 0.05). Interesting, the result of the expression of autophagy-related factor proteins *in vivo* were consistent with those of mRNA expression (Figures 5D–F). The results showed that the LC3 and Beclin-1 protein expression levels in the ischemic hemisphere brain were significantly higher (*p* < 0.01, Figures 5D,E), whereas the protein expression of P62 was significantly lower in the ischemic hemisphere brain than in the sham group (*p* < 0.01, Figure 5F). 120 mg/kg OMT posttreatment can reverse the above protein expression.

### OMT Inhibited Autophagy by Activating PI3K/Akt/mTOR Pathway

**FIGURE 6:**
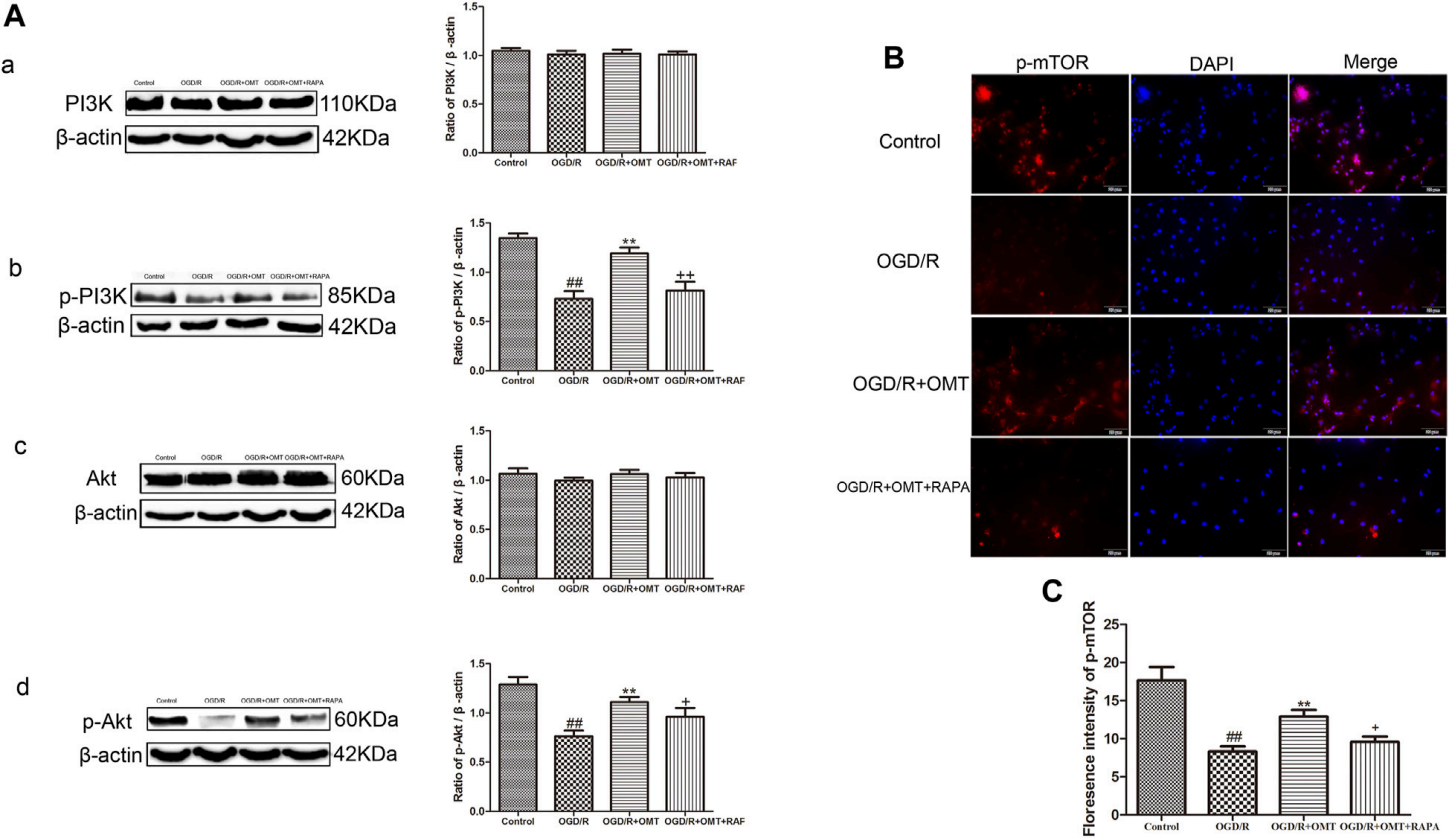
Assessment of the expression levels of (*p*)-PI3K (*p*)-Akt, and p-mTOR in the ischemic hemisphere in the neonatal rats at 24 h after oxygen-glucose deprivation reperfusion (OGD/R). **(A)** Representative Western blots and quantitative analyses show the protein expression of **(A**, **B)** PI3K and p-PI3K, and **(C**, **D)** Akt and p-Akt. **(B)** Immunofluorescent-stained p-mTOR in the primary hippocampal neurons at 24 h after OGD/R. **(C)** Analysis of fluorescence intensity of p-mTOR. The results are expressed as mean ± SEM (*n* = 6 per group). ^##^ *p* < 0.01 vs. the sham group; *p < 0.05, **p < 0.01 vs. the HI group; and ^+^ *p* < 0.05 vs. the oxymatrine (OMT) group.

These above experiments result preliminarily suggested that OMT could improve HIBD in neonatal rats via regulating autophagy-related protein. To further examine the relationship between the regulative effects of OMT on neuronal autophagy and the PI3K/Akt/mTOR pathway in HIBD, we detected the expression of PI3K, p-PI3K, Akt, *p*-Akt, and *p*-mTOR by Western blot and immunofluorescence analyses (Figures 5, 6). The expression of PI3K, Akt, and mTOR were significantly reduced in the HI group than that in the sham group. Post-administration with OMT could reverse the expression of these kinases (*p* < 0.01). The expression of p-PI3K and p- Akt remained a basal expression under normal condition. OMT significantly enhanced the levels of p-PI3K and *p*-Akt relative to those in the OGD/R group (*p* < 0.01) but no statistically significant difference was observed in the expression of t-PI3K and t-Akt in the above groups (*p* > 0.05, Figure 6). Such rise in levels was inhibited by RAPA in the OMT + RAPA group relative to that in the OMT group (*p* < 0.01, *p* < 0.05 and *p* < 0.05, Figures 6A,C).

## Discussion

OMT protected against brain injury in a neonatal rat model of HIBD by improving the early neurologic impairments, infarct volume, brain water volume, and neuronal damage and degeneration. OMT also exhibited also neuroprotective effects and reduced OGD/R-mediated cell death and the increase of mPTP in the primary hippocampal neurons. OMT decreased the formation of autophagosome and reversed the levels of autophagy-related factor expression induced by HIBD and OGD/R. OMT promoted the activation of PI3K and Akt and increased the fluorescence intensity of *p*-mTOR under HI. RAPA, an mTOR inhibitor, inhibited the mTOR expression and partially counteracted the neuroprotective effects of OMT. These findings suggest that OMT played a neuroprotective role in treating HIBD, and the underlying mechanisms may have partly involved the activation of the PI3K/AKT/mTOR signaling pathway.

HIBD is the most common clinical disease of newborn that seriously threatens the physical and mental health of fetus and newborn. Animal model is of great significance to study the pathological mechanism of disease and evaluate the effect of therapeutic intervention. In the present study, we utilized a modified Rice-Vannucci model because the degree of development of the brain tissues of 7-day-old newborn rats is approximately equivalent to that of human fetuses or newborns because both species are in the synapse formation (Dobbing and Sands, 1979; McDonald and Johnston, 1990). In addition, the secondary pathological changes induced by HI in the rat model are similar to those of asphyxiated newborns. The latter is widely used to study neonatal cerebral ischemia–hypoxia injury. Cerebral neurons are extremely vulnerable to HI, because the metabolism of the immature brain is relatively vigorous, and their oxygen consumption accounts for approximately 50% of the total oxygen consumption. These neurons lack the necessary enzyme for effective glycolysis (Li et al., 2017). Once the oxygen and blood in the brain are blocked, which lead to a sharp lack of energy, depolarization of cell membranes occurs, followed by neuronal death. Eventually, a series of lesions, such as edema and infarction, are presented.

It has been reported in the literature that HIBD is accompanied by short-term neurological dysfunction. As expected, we observed that the latencies of the righting reflex, negative geotaxis reflex and cliff avoidance reflex remarkably prolonged subjected to HI in current study. Administering OMT improved these reflexes, revealing that OMT can improve early neurological dysfunction induced by HI in neonatal rats.

Brain edema is a common pathological state of brain tissue under HI. Current studies tend to focus on the cytotoxic brain edema and vasculogenic brain edema (Igor, 1987), which have many similarities in the mechanism of HIBD. Therefore, brain edema can be used as an indirect index to evaluate brain injury. In the present study, 120 mg/kg OMT could significantly ameliorate cerebral water content, which suggested that OMT protected against HIBD by mitigating cerebral water volume. In addition, the HE and TUNEL staining can provide further evidence of the protective effect of OMT.

HI can cause neuronal damage and death. The hippocampus and cortex are the most sensitive regions reflecting HI (Cheng et al., 2012), so we choose these areas to observe neuronal damage and morphological changes. The HE staining results showed that the cell density significantly decreased, became arranged loosely, and were stained unevenly. In addition, a number of vacuoles appeared in cerebral cortex, hippocampal CA3, and CA1 in the HI group compared with the sham group. After intervention with OMT, these symptoms were alleviated. In addition, TUNEL staining results showed that a large number of TUNEL-positive neurons in the ischemic cortex, while OMT treatment promoted the recovery of the injured neurons. These results indicated that OMT alleviated the pathological damage and death of neurons induced by HI.

We also established the OGD/R model *in vivo* to evaluate the cell viability and damage using the MTT method and mitochondrial permeability transition pore (mPTP) assays. The increase in ROS and calcium overload caused by HI could lead to the opening of the mPTP, the disorder of mitochondrial membrane potential, decoupling of respiratory chain, and blockage of ATP synthesis. These processes eventually lead to the swelling, rupture, and death of the mitochondria. Therefore, the degree of neuronal injury is related to the opening of mPTP (Halestrap, 2009). In our current research, OMT attenuated OGD/R-induced neuronal death and inhibited the increase of intracellular mPTP. These results indicate that OMT had a protective effect on the OGD/R-induced hippocampal neuronal damage. Brain infract volume is generally considered as a standard indicator for evaluating the effects of drugs on brain ischemic. RAPA intervention could reverse the remission effect of OMT on the cerebral infarction and neuronal damage induced by HIBD. These results suggest that the protective effect of OMT on HIBD in neonatal rats may be related to the mTOR pathway.

HI cause three types of cell death, namely, apoptosis, necrosis, and autophagy. Autophagy is a precise and orderly process that allows the degradation and recycling of cellular organelles and misfolded proteins under physiological conditions. Several studies have indicated that autophagy plays a significant role in HIBD (Northington et al., 2011), that is, an overactivated autophagy leads to rapid neuronal death (Levine and Yuan, 2005; Koike et al., 2008). Electron microscopy is currently considered to be a gold marker for the formation of autophagosome in tissues, and monodansylcadaverine (MDC) is an eosinophilic fluorescent stain, usually used to detect specific markers for the formation of autophagosome in cells. The results showed that after OMT treatment, the formation level of the autophagosome decreased significantly, and the regulatory effect of OMT on autophagosome formation was partially blocked by the administration of RAPA. The results further suggest that OMT played an antiexcessive autophagy role through the mTOR signaling pathway and participated in maintaining the stability of neurons and the recovery of injury. This process also suggested that regulating the level of autophagy may be one of the roles of OMT in protecting HIBD.

The PI3K/Akt/mTOR pathway is an intracellular signaling pathway involved in regulating cell survival and death. Moreover, as the only autophagic protective pathway, it plays an important role in HIBD (Yu et al., 2018). The mTOR, which belongs to the PI3K protein kinase family, is sensitive to RAPA and participates in regulating cell growth, apoptosis and autophagy (Hu et al., 2015). PI3K/Akt is one of the key upstream regulators of mTOR and plays an important role in regulating mTOR (Gunn and Hailes, 2008). Subsequently, when activated by PI3K, Akt could phosphorylate TSC2, dissociate the TSC1/TSC2 complex and activate mTOR (Pankiv et al., 2007). Finally, mTOR regulated the expression of downstream autophagy-related proteins Beclin1, LC3 and P62 and participated in the regulation of autophagy. LC3 is located on the autophagosome membrane and plays an important role in the formation of complete autophagosome. Beclin-1 plays an important role in lysosome fusion and autophagy formation in autophagy, and is another important autophagy marker. As an index of autophagic flow, some studies have shown that the level of P62 increases and the level of LC3 decreases, indicating that the autophagic flow is complete (Bockaert and Marin, 2015). Some reports have also demonstrated that promoting mTOR activation can play a neuroprotective role in HIBD (Chen et al., 2012). To further explore the potential protective mechanism of OMT in neonatal rats and primary hippocampal neurons after HI, we verified the expression of PI3K, p-PI3K, Akt, p-Akt, p-mTOR and autophagy-related factors. Consistent with a previous study, the present results showed that the expression of PI3K, Akt, mTOR and P62 in the brain tissue was significantly decreased after HI, while protein expression of LC3 and Beclin-1increased. After the intervention with OMT, the protein expression was significantly reversed. These results suggest that OMT can alleviate the damage of HIBD neurons by up-regulating the expression of PI3K, Akt, mTOR and P62, and down-regulating the expression of LC3 and Beclin-1. At the cellular level, the expression of p-PI3K, *p*-Akt, *p*-mTOR, and P62 significantly decreased, and the expression of LC3 and Beclin-1 significantly increased compared with that of the control group. OMT could significantly reverse the activity of pathway-related factors, reduce the expression of Beclin1 and LC3, increase the expression of P62 and improve the HIBD in neonatal rats. However, in the mTOR inhibitor treatment group, RAPA partially eliminated the effects of OMT on the expression levels of p-PI3K, *p*-Akt, *p*-mTOR, LC3, Beclin-1 and P62. These findings confirm that the neuroprotective effect of OMT on HIBD may be related to the regulation of the PI3K/Akt/mTOR signaling pathway and the inhibition of excessive autophagy. However, besides the PI3K/Akt/mTOR pathway, other factors may be involved in the neuroprotective effect induced by OMT, and related factors need further study.

In conclusion, by performing *in vivo* and *in vitro* experiments, we demonstrated that posttreatment with OMT efficaciously exerted neuroprotective effect against HIBD in the neonatal rats. The underlying mechanisms may include the regulation of the PI3K/Akt/mTOR signaling pathway and inhibition of excessive autophagy of neurons. However, our study had some limitations. First, we did not explore different times to evaluate the optimal therapeutic window of OMT for the neonatal HIBD. Second, accruing studies have shown sex-specific differences in the HIBD model. Further studies are required to explore the differences in the effects of OMT on the HIBD in female and male neonatal rats.

## Data Availability

The original contributions presented in the study are included in the article/Supplementary Material, further inquiries can be directed to the corresponding authors.

## References

[B1] Bockaert J. Marin P. (2015). mTOR in brain physiology and pathologies. Physiol. Rev. 95 (4), 1157–1187. 10.1152/physrev.00038.2014 26269525

[B2] Bodine S. C. Stitt T. N. Gonzalez M. Kline W. O. Stover G. L. Bauerlein R. (2001). Akt/mTOR pathway is a crucial regulator of skeletal muscle hypertrophy and can prevent muscle atrophy *in vivo* . Nat. Cell Biol 3 (11), 1014–1019. 10.1038/ncb1101-1014 11715023

[B3] Chen H. Xiong T. Qu Y. Zhao F. Ferriero D. Mu D. (2012). mTOR activates hypoxia-inducible factor-1α and inhibits neuronal apoptosis in the developing rat brain during the early phase after hypoxia-ischemia. Neurosci. Lett. 507 (2), 118–123. 10.1016/j.neulet.2011.11.058 22178140 PMC3525671

[B4] Cheng O. Li Z. Han Y. Jiang Q. Yan Y. Cheng K. (2012). Baicalin improved the spatial learning ability of global ischemia/reperfusion rats by reducing hippocampal apoptosis. Brain Res. 1470, 111–118. 10.1016/j.brainres.2012.06.026 22796597

[B5] Cui L. Zhang X. Yang R. Wang L. Liu L. Li M. (2011). Neuroprotection and underlying mechanisms of oxymatrine in cerebral ischemia of rats. Neurol. Res. 33 (3), 319–324. 10.1179/016164110x12759951866876 20626959

[B6] Dobbing J. Sands J. (1979). Comparative aspects of the brain growth spurt. Early Hum. Dev. 3 (1), 79–83. 10.1016/0378-3782(79)90022-7 118862

[B7] Dong X. Q. Du Q. Yu W. H. Zhang Z. Y. Zhu Q. Che Z. H. (2013). Anti-inflammatory effects of oxymatrine through inhibition of nuclear factor-kappa B and mitogen-activated protein kinase activation in lipopolysaccharide-induced BV2 microglia cells. Iran J. Pharm. Res. 12 (1), 165–174. 24250585 PMC3813192

[B8] Douglas-Escobar M. Weiss M. D. (2015). Hypoxic-ischemic encephalopathy. JAMA Pediatr. 169, 397–403. 10.1001/jamapediatrics.2014.3269 25685948

[B9] Edwards A. D. Brocklehurst P. Gunn A. J. Halliday H. Juszczak E. Levene M. (2010). Neurological outcomes at 18 months of age after moderate hypothermia for perinatal hypoxic ischaemic encephalopathy: synthesis and meta-analysis of trial data. BMJ 340, c363. 10.1136/bmj.c363 20144981 PMC2819259

[B10] Funaya N. Haginaka J. (2012). Matrine- and oxymatrine-imprinted monodisperse polymers prepared by precipitation polymerization and their applications for the selective extraction of matrine-type alkaloids from *Sophora* flavescens Aiton. J. Chromatogr. A 1248, 18–23. 10.1016/j.chroma.2012.05.081 22695694

[B11] Gunn R. M. Hailes H. C. (2008). Insights into the PI3-K-PKB-mTOR signalling pathway from small molecules. J. Chem. Biol. 1 (1–4), 49–62. 10.1007/s12154-008-0008-0 19568798 PMC2698322

[B12] Halestrap A. P. (2009). What is the mitochondrial permeability transition pore?. J. Mol. Cell Cardiol. 46 (6), 821–31. 10.1016/j.yjmcc.2009.02.021 19265700

[B13] Hu Z. Yang B. Mo X. Xiao H. (2015). Mechanism and regulation of autophagy and its role in neuronal diseases. Mol. Neurobiol. 52 (3), 1190–1209. 10.1007/s12035-014-8921-4 25316381

[B14] Huang L. Chen C. Zhang X. Li X. Chen Z. Yang C. (2018). Neuroprotective effect of curcumin against cerebral ischemia-reperfusion via mediating autophagy and inflammation. J. Mol. Neurosci. 64 (1), 129–139. 10.1007/s12031-017-1006-x 29243061

[B15] Igor K. (1987). Pathophysiological aspects of brain edema. Acta Neuropathol. 72 (3), 236–239. 10.1007/BF00691095 3564903

[B16] Jacobs S. E. Berg M. Hunt R. Tarnow-Mordi W. O. Inder T. E. Davis P. G. (2013). Cooling for newborns with hypoxic ischaemic encephalopathy. Cochrane Database Syst. Rev. 31, CD003311. 10.1002/14651858.CD003311.pub3 14583966

[B17] Koh S.-H. Lo E. H. (2015). The role of the PI3K pathway in the regeneration of the damaged brain by neural stem cells after cerebral infarction. J. Clin. Neurol. 11 (4), 297–304. 10.3988/jcn.2015.11.4.297 26320845 PMC4596106

[B18] Koike M. Shibata M. Tadakoshi M. Gotoh K. Komatsu M. Waguri S. (2008). Inhibition of autophagy prevents hippocampal pyramidal neuron death after hypoxic-ischemic injury. Am. J. Pathol. 172 (2), 454–469. 10.2353/ajpath.2008.070876 18187572 PMC2312361

[B19] Lawn J. E. Cousens S. Zupan J. (2005). 4 million neonatal deaths: when? Where? Why?. The Lancet 365 (9462), 891–900. 10.1016/s0140-6736(05)71048-5 15752534

[B20] Levine B. Yuan J. (2005). Autophagy in cell death: an innocent convict?. J. Clin. Invest. 115 (10), 2679–2688. 10.1172/jci26390 16200202 PMC1236698

[B21] Li D. Luo L. Xu M. Wu J. Chen L. Li J. (2017). AMPK activates FOXO3a and promotes neuronal apoptosis in the developing rat brain during the early phase after hypoxia-ischemia. Brain Res. Bull. 132, 1–9. 10.1016/j.brainresbull.2017.05.001 28499802

[B22] Li Y. Wang X. Cai C Zeng S. Bai J. Guo K. (2019). FGF21 promotes functional recovery after hypoxic-ischemic brain injury in neonatal rats by activating the PI3K/Akt signaling pathway via FGFR1/β-klotho. Exp. Neurol. 317, 34–50. 10.1016/j.expneurol.2019.02.013 30802446

[B23] Lin T. N. He Y. Y. Wu G. Khan M. Hsu C. Y. (1993). Effect of brain edema on infarct volume in a focal cerebral ischemia model in rats. Stroke 24 (1), 117–121. 10.1161/01.str.24.1.117 8418534

[B24] Liu H. Sun X. Gong X. Wang G. (2019). Human umbilical cord mesenchymal stem cells derived exosomes exert antiapoptosis effect via activating PI3K/Akt/mTOR pathway on H9C2 cells. J. Cell Biochem. 120 (9), 14455–14464. 10.1002/jcb.28705 30989714

[B25] Martin A. Karina Z. Floris G. Bell F. V. Peeters-Scholte C. P. (2019). Neuroprotective strategies following perinatal hypoxia-ischemia: taking aim at NOS. Free Radic. Biol. Med. 142, 123–131. 10.1016/j.freeradbiomed.2019.02.025 30818057

[B26] McDonald J. W. Johnston M. V. (1990). Physiological and pathophysiological roles of excitatory amino acids during central nervous system development. Brain Res. Rev. 15 (1), 41–70. 10.1016/0165-0173(90)90011-c 2163714

[B27] Northington F. J. Chavez-Valdez R. Martin L. J. (2011). Neuronal cell death in neonatal hypoxia-ischemia. Ann. Neurol. 69 (5), 743–758. 10.1002/ana.22419 21520238 PMC4000313

[B28] Pankiv S. Clausen T. H. Lamark T. Brech A. Bruun J.-A. Outzen H. (2007). p62/SQSTM1 binds directly to Atg8/LC3 to facilitate degradation of ubiquitinated protein aggregates by autophagy. J. Biol. Chem. 282, 24131–24145. 10.1074/jbc.m702824200 17580304

[B29] Panthati M. K. Rao K. N. V. Sandhya S. David B. (2012). A review on phytochemical, ethnomedical and pharmacological studies on genus *Sophora* . Fabaceae. Panthati Murali Krishna 22 (5), 1145–1154. 10.1590/S0102-695X2012005000043

[B30] Vannucci R. C. Vannucci S. J. (2005). Perinatal hypoxic-ischemic brain damage: evolution of an animal model. Dev. Neurosci. 27 (2-4), 81–86. 10.1159/000085978 16046840

[B31] Wang J. Zhang D. Fu X. Lu Z. Guo Y. Liu X. (2018). Carbon monoxide-releasing molecule-3 protects against ischemic stroke by suppressing neuroinflammation and alleviating blood-brain barrier disruption. J. Neuroinflam 15 (1), 188. 10.1186/s12974-018-1226-1 PMC6014004 29929562

[B32] Wang S.-B. Jia J.-P. (2014). Oxymatrine attenuates diabetes-associated cognitive deficits in rats. Acta Pharmacol. Sin 35 (3), 331–338. 10.1038/aps.2013.158 24442148 PMC4647892

[B33] Wei W. Lan X. B. Liu N. Yang J. M. Du J. Ma L. (2019). Echinacoside alleviates hypoxic-ischemic brain injury in neonatal rat by enhancing antioxidant capacity and inhibiting apoptosis. Neurochem. Res. New York. 44 (7), 1582–1592. 10.1007/s11064-019-02782-9 30911982

[B34] Wu W. Wei W. Lu M. Zhu X. Liu N. Niu Y. (2017). Neuroprotective effect of chitosan oligosaccharide on hypoxic-ischemic brain damage in neonatal rats. Neurochem. Res. 42 (11), 3186–3198. 10.1007/s11064-017-2356-z 28755288

[B35] Wu Y. W. Gonzalez F. F. (2015). Erythropoietin: a novel therapy for hypoxic-ischaemic encephalopathy?. Dev. Med. Child. Neurol. 57 (Suppl. 3), 34–39. 10.1111/dmcn.12730 25800490

[B36] Yu Y. Wu X. Pu J. Luo P. Ma W. Wang J. (2018). Lycium barbarum polysaccharide protects against oxygen glucose deprivation/reoxygenation-induced apoptosis and autophagic cell death via the PI3K/Akt/mTOR signaling pathway in primary cultured hippocampal neurons. Biochem. Biophys. Res. Commun. 495 (1), 1187–1194. 10.1016/j.bbrc.2017.11.165 29183728

[B37] Yıldız E. P. Ekici B. Tatlı B. (2017). Neonatal hypoxic ischemic encephalopathy: an update on disease pathogenesis and treatment. Expert Rev. Neurother. 17 (5), 449–459. 10.1080/14737175.2017.1259567 27830959

[B38] Zhao P. Zhou R. Li H. N. Yao W. X. Qiao H. Q. Wang S. J. (2015). Oxymatrine attenuated hypoxic-ischemic brain damage in neonatal rats via improving antioxidant enzyme activities and inhibiting cell death. Neurochem. Int. 89, 7–27. 10.1016/j.neuint.2015.06.008 26120022

[B39] Zhao P. Chang R.-Y. Liu N. Wang J. Zhou R. Qi X. (2018). Neuroprotective effect of oxysophocarpine by modulation of MAPK pathway in rat hippocampal neurons subject to oxygen-glucose deprivation and reperfusion. Cell Mol Neurobiol. 38 (2), 529–540. 10.1007/s10571-017-0501-5 28488010 PMC11481923

[B40] Zhu W. Gao Y. Wan J. Lan X. Han X. Zhu S. (2018). Changes in motor function, cognition, and emotion-related behavior after right hemispheric intracerebral hemorrhage in various brain regions of mouse. Brain Behav. Immun. 69, 568–581. 10.1016/j.bbi.2018.02.004 29458197 PMC5857479

